# A multimodal MRI study of the neural mechanisms of emotion regulation impairment in women with obesity

**DOI:** 10.1038/s41398-019-0533-3

**Published:** 2019-08-20

**Authors:** Trevor Steward, Maria Picó-Pérez, Gemma Mestre-Bach, Ignacio Martínez-Zalacaín, Maria Suñol, Susana Jiménez-Murcia, Jose A. Fernández-Formoso, Nuria Vilarrasa, Amador García-Ruiz-de-Gordejuela, Misericordia Veciana de las Heras, Nuria Custal, Nuria Virgili, Rafael Lopez-Urdiales, José M. Menchón, Roser Granero, Carles Soriano-Mas, Fernando Fernandez-Aranda

**Affiliations:** 1 Department of Psychiatry, Bellvitge University Hospital-IDIBELL, C/Feixa Llarga s/n, 08907 Barcelona, Spain; 2 Ciber Fisiopatología Obesidad y Nutrición (CIBERObn), Instituto Salud Carlos III, C/Feixa Llarga s/n, 08907 Barcelona, Spain; 3 Melbourne School of Psychological Sciences, Faculty of Medicine, Dentistry and Health Sciences, University of Melbourne, Parkville, VIC 3010 Australia; 4 Life and Health Sciences Research Institute (ICVS), School of Medicine, University of Minho, Braga, Portugal; 5 ICVS/3B’s—PT Government Associate Laboratory, Braga/Guimarães, Portugal; 6 Department of Clinical Sciences, School of Medicine, University of Barcelona, C/Feixa Llarga s/n, 08907 Barcelona, Spain; 7 Ciber Salud Mental (CIBERSAM), Instituto Salud Carlos III, C/Feixa Llarga s/n, 08907 Barcelona, Spain; 8 Department of Endocrinology and Nutrition, University Hospital of Bellvitge-IDIBELL, C/Feixa Llarga s/n, 08907 Barcelona, Spain; 9 CIBERDEM-CIBER de Diabetes y Enfermedades Metabólicas Asociadas, Instituto de Salud Carlos III, C/Feixa Llarga s/n, 08907 Barcelona, Spain; 10 Bariatric and Metabolic Surgery Unit, Service of General and Gastrointestinal Surgery, Bellvitge University Hospital-IDIBELL, C/Feixa Llarga s/n, 08907 Barcelona, Spain; 11 Neurology Department, Bellvitge University Hospital-IDIBELL, C/Feixa Llarga s/n, 08907 Barcelona, Spain; 12 Departament de Psicobiologia i Metodologia de les Ciències de la Salut, Universitat Autònoma de Barcelona, 08193 Barcelona, Spain

**Keywords:** Psychiatric disorders, Neuroscience, Human behaviour

## Abstract

Maladaptive emotion regulation contributes to overeating and impedes weight loss. Our study aimed to compare the voluntary downregulation of negative emotions by means of cognitive reappraisal in adult women with obesity (OB) and female healthy controls (HC) using a data-driven, multimodal magnetic resonance imaging (MRI) approach. Women with OB (*n* = 24) and HC (*n* = 25) carried out an emotion regulation task during functional MRI scanning. Seed-to-voxel resting-state connectivity patterns derived from activation peaks identified by this task were compared between groups. Diffusion tensor imaging (DTI) was used to examine white matter microstructure integrity between regions exhibiting group differences in resting-state functional connectivity. Participants in the OB group presented reduced activation in the ventromedial prefrontal (vmPFC) cortex in comparison to the HC group when downregulating negative emotions, along with heightened activation in the extrastriate visual cortex (*p* < 0.05, AlphaSim-corrected). Moreover, vmPFC peak activity levels during cognitive reappraisal were negatively correlated with self-reported difficulties in emotion regulation. OB patients exhibited decreased functional connectivity between the vmPFC and the temporal pole during rest (peak-pFWE = 0.039). Decreased fractional white-matter track volume in the uncinate fasciculus, which links these two regions, was also found in participants with OB. Taken together, our findings are indicative of emotion regulation deficits in OB being underpinned by dysfunctional hypoactivity in the vmPFC and hyperactivity in the extrastriate visual cortex. Our results provide a potential target circuit for neuromodulatory interventions to improve emotion regulation skills and weight-loss intervention outcomes.

## Introduction

Obesity (OB) has become a major global health challenge^1^ and a significant contributor to a reduction in improving mortality rates in the United States^2^. Emotional distress is increasingly understood as one of the root causes of weight gain^3^ and recent research found affective negative states to be a predictor of lapses during a 12-month weight-loss program^4^.

Emotion regulation pertains to the implementation of a conscious or nonconscious strategy to start, stop, or otherwise modulate the trajectory of an emotion^5^. When compared with control groups, meta-analyses have demonstrated that individuals with OB are more likely to endorse higher scores of alexithymia, difficulty in identifying feelings, and an externally oriented thinking style, which, in turn, may interfere with subsequent emotion regulation responses^6^. Relatedly, a stressful environment in combination with ineffective emotion regulation has been linked to abnormal cortisol patterns^7^, emotional eating^8^, a sedentary lifestyle^9^, and sleep problems^10^. Indeed, the latest models of OB have stressed the need to incorporate emotion regulation training into interventions in order to improve treatment outcomes^11–13^.

Based on the findings of numerous neuroimaging studies, mechanistic neural models of emotion regulation coincide in highlighting the role of prefrontal and cingulate control systems in modulating activity in perceptual, semantic, and affect systems^14^. For example, one pathway involved in the downregulation of negative emotions consists of the recruitment of dorsomedial or ventrolateral prefrontal regions so as to diminish amygdala responses via their impact on the ventromedial prefrontal cortex (vmPFC^15,16^). Other studies using functional magnetic resonance imaging (fMRI) in patients with psychiatric disorders, including those with disordered eating, have described abnormal neural activation patterns encompassing, among others, the above-mentioned regions during emotion regulation tasks^17,18^. Likewise, the only fMRI study to date using a cognitive reappraisal task in individuals with OB found that, when instructed to regulate negative emotions by means of cognitive reappraisal, young adults with excess weight displayed heightened activation in the right anterior insula, as well as decreased functional coupling between the right anterior insula and dorsal regions of the prefrontal cortex^19^. However, further research efforts are needed to fully characterize the neurobiological underpinnings of altered emotion regulation in OB.

In parallel, current MRI assessments grant researchers the ability to delineate the neurobiological correlates of behavior in multiple units of analysis, which, when properly integrated, permit a deeper understanding of the mechanistic effects accounting for disrupted behaviors. This has led to the combination of different imaging methods in order to investigate the relationship between task-related local brain activations and network-level connectivity assessments, as well as between functional and structural measurements^20^. Within the context of OB, one multimodal study obtained results supporting an association between higher body mass index (BMI) and decreased fractional anisotropy (FA) in white matter fibers connecting brain regions that support working memory, though no link with blood-oxygen-level dependent (BOLD) activity was found^21^. This finding may indicate that changes in brain structure precede deviations in function. Other studies have opted to combine both intrinsic resting-state and task-based activations, identifying alterations in the middle frontal gyrus and occipital areas during perceptual processes that may be explained by diminished functional integration^22^. Overall, these results uphold the benefits of utilizing multimodal imaging approaches to reveal the neurobiological abnormalities underpinning the behaviors found in OB.

Taking into account the relevance of emotion regulation in OB interventions^23^, the aim of the present study was to use multimodal MRI methods to examine emotion regulation in adult women with OB and healthy weight controls. As opposed to selecting which brain regions to explore a priori, the study at hand employed a data-driven approach in which the findings obtained from the cognitive reappraisal paradigm informed where the subsequent MRI modality would be used. Moreover, we sought to investigate associations between fMRI activation patterns and the structural properties of white matter fiber tracts with self-reported emotion regulation deficits and anthropometric variables.

## Materials/subjects and methods

### Participants

Our sample was made up of 24 adult women with OB recruited from the Bariatric and Metabolic Surgery Unit and the Endocrinology and Nutrition Unit at Bellvitge University Hospital (Barcelona, Spain). Participants with OB were compared to 25 healthy-weight controls (HC) from the same hospital catchment area. As part of the evaluation procedure, all participants underwent the Mini-International Neuropsychiatric Interview (M.I.N.I.) with staff psychologists from the Department of Psychiatry at Bellvitge University Hospital^24^. Complete inclusion and exclusion criteria for this are included in the Supplementary Information. Patients exhibiting binge-eating behavior were not included in the study sample.

The present study was carried out in accordance with the latest version of the Declaration of Helsinki. The Bellvitge University Hospital Clinical Research Ethics Committee and Institutional Review Board approved the study (PR146/14). Signed informed consent was obtained from all participants.

### Clinical measures

All participants completed the Difficulties in Emotion Regulation Scale (DERS) (Spanish validation by Wolz et al. ^25^). This 36-item self-report measure assesses emotion regulation difficulties using six separate subscales. Higher scores on the DERS indicate greater emotion regulation impairment. Cronbach’s alphas to determine the internal consistency of the DERS subscales are included in Table S1 of the Supplementary Information.

### Anthropometric measures

A Tanita BC-420MA was utilized to assess body composition and to calculate BMI. This noninvasive and validated device (Tanita BC-420MA, Tanita Corp., Tokyo, Japan) uses bioelectrical impedance analysis to measure weight and body composition variables (i.e. body fat percentage^26^). Height was measured via a stadiometer.

### Statistical analyses involving clinical and anthropometric measures

Statistical analysis was carried out with SPSS 23 (IBM Corp., Armonk, NY, USA). Between-group comparisons were carried out using independent sample *t*-tests, and linear associations were estimated using Pearson’s correlations. Finner’s method was used to control for Type-I error stemming from multiple comparisons^27^, Levene’s test assessed the equality of variances between the groups, and Shapiro–Wilk tests were performed to confirm normality. Effect size for mean differences was measured using Cohen’s-*d* coefficient (|*d*| > 0.2–0.5 was considered low, |*d*| > 0.5–0.8 moderate, and |*d*| > 0.8 large^28^. For Pearson’s correlations, and due to the strong association between this coefficient and sample size, significance was based on effect size (|*r*| > 0.10–0.24 was considered low, |*r*| > 0.24–0.37 moderate, and |*r*| > 0.37 large)^29^. Estimated power analysis for mean comparison *t*-tests are reported in Table S1, whereas estimated power for the one-sample correlation tests were between 0.60 and 0.95.

### fMRI cognitive reappraisal task

See the Supporting Information for additional details on MRI image acquisition and preprocessing.

A modified version of the cognitive reappraisal task designed by Phan et al.^30^ was used to evaluate emotion regulation during fMRI scanning. This task has been used in different psychiatric populations^31^ and in patients with excess weight^19^. The task consists of presenting a series of blocks displaying neutral or negative picture stimuli that participants were instructed to (1) “Observe” (to passively observe neutral images); (2) “Maintain” (to actively sustain the negative emotions elicited by the images); or (3) “Regulate” (to reappraise and reduce the intensity of negative emotions by means of previously trained cognitive reappraisal techniques). Further descriptions of the task have been reported elsewhere^30^ and can be found in the Supporting Information.

### fMRI emotion regulation task effects

#### First-level analyses

The two contrasts of interest defined for first-level (single-subject) analysis were Maintain vs. Observe and Regulate vs. Maintain. The former assesses brain activations associated with negative emotion generation, whereas the latter delineates brain activations associated with cognitive reappraisal^18,32^. Conditions were modeled for the 20 s that the images were on the screen and did not include instruction and rating periods. The BOLD response at each voxel was convolved with the SPM12 canonical hemodynamic response function using a 128-s high-pass filter.

#### Second-level analyses

Between-group comparisons in task-induced activations were conducted via two-sample *t*-tests using group (HC vs. OB) as the main factor. Analyses were carried out using masks generated by extracting and conjoining areas from one-sample (OB and HC) activations for each contrast. The peak activation differences derived were extracted and entered into an SPSS data matrix to assess their relationship with clinical and anthropometric measures.

#### Psychophysiological interactions (PPI) analysis

In order to explore differences in task-induced connectivity between the brain regions activated during the emotion regulation task, PPI analyses were conducted using SPM 12. Here, we explored the impact of the contrast of interest (the ‘psychological’ factor) on the strength of time-course correlations between our empirically obtained region of interest (ROI, the ‘physiological’ factor) with the conjoining areas from one-sample activations. To perform first-level analyses, ROIs were drawn from the regions showing group differences during the emotion regulation task. Specifically, the following regions stemming from the results of the Regulate>Maintain contrast of the emotion regulation task (see below) were used as seeds: the left vmPFC (*x* = −10, *y* = 64, *z* = −18), and the right extrastriate visual cortex (*x* = 56, *y* = −72, *z* = 10), which were defined with 3-mm radial spheres (see Table 2). Functional connectivity maps were estimated for the selected seeds by including our signal of interest (seed) in interaction with the task blocks, controlling for the raw signal of the seed and the task blocks. Resulting images were then included in a two-sample *t*-test model for each seed (second-level) to assess between-group effects.

#### Significance thresholding

Statistical significance was determined by a combination of voxel-level and cluster-extent thresholds using the AlphaSim algorithm as implemented in the SPM RESTplus V1.2 toolbox^33^. The minimum spatial cluster extent (KE) to satisfy a family-wise error (FWE) rate correction of pFWE < 0.05 varied over the different contrasts. Input parameters to AlphaSim included a voxel-level probability of *p* < 0.001, a rmm (edge connected for cluster) of 5, a full width at half maximum (FWHM) corresponding to the actual smoothing of the data after model estimation, and a mask volume contingent on the contrast of interest. As a result, for task activation analyses we obtained a cluster-extent threshold of 33 voxels and thresholds of 10 and 51 voxels for PPI analyses to satisfy a pFWE < 0.05.

### Resting-state analysis

CONN Toolbox (Version 17, McGovern Institute for Brain Research, Massachusetts Institute of Technology, Cambridge, USA, http://www.nitrc.org/projects/conn) was used for resting-state, seed-to-voxel connectivity analyses. A band-pass filter with cut-off frequencies of 0.008 and 0.09 Hz was applied to exclude drifts and high-frequency activations. ROIs were generated as 5-mm radius spheres centered on the peak coordinates from significant task activations acquired from the emotion regulation task (i.e. the vmPFC and the right extrastriate visual cortex). Whole-brain functional connectivity maps were estimated for these two seeds, and resulting images were included in second-level two-sample *t*-tests to assess between-group effects.

### Diffusion-weighted imaging (DWI) analyses

DWI data were visually inspected for motion and dropout slices, with individual volumes discarded (subjects were excluded when <33 volumes remained). The DIFFPREP module in TORTOISE V3.1.0^34^ was used to compute distortion corrections for subject motion, eddy currents, and basic EPI distortions. Fat_proc* programs in AFNI were used to invert the contrast of the T1w images to imitate T2-weighted images in order to provide an anatomical reference volume within TORTOISE^35^. Lastly, preprocessed DWIs were exported to AFNI for diffusion tensor (DT) and associated parameter fitting.

#### Target placement

Pairs of regions displaying significant between-group differences in intrinsic resting-state functional connectivity were selected as targets for tractography analysis given that previous studies have established that positive relationships between BOLD signal and white matter measures are largely found in resting state data^36^. One pair of targets was placed within the left vmPFC and the left temporal pole. The other included the right extrastriate visual cortex and the left inferior temporal lobe. These targets were generated in MNI space after which they were transformed to each subject’s DW space and inflated in volume to ensure adequate WM coverage.

#### Probabilistic tractography

White-matter regions of interest (referred to hereafter as WM-ROIs) connecting pairs of resting-state targets were identified using probabilistic tractography, as implemented in AFNI’s FATCAT utility toolkit^37^. FATCAT efficiently finds white-matter connections within networks and provides quantitative measures for all identified WM-ROIs. DTI parameter (FA and the direction of the first eigenvector of the DTI tensor relative to the mean direction) uncertainty maps for probabilistic tracking were calculated with FATCAT 3dDWUncert using 50 iterations^37^. Parameters for probabilistic tracking were five seed points per voxel, 1000 Monte Carlo iterations, and a threshold fraction of 0.001, and, at each iteration, the algorithm found locations of tracts connecting pairs of targets. Only white-matter tracts connecting targets in at least 85% of the total sample were included. Standard propagation parameters for this algorithm were used: 60° maximum angle of propagation confined to voxels with FA > 0.2. Here we report FA and mean diffusivity (MD) for each WM-ROI, as calculated by FATCAT, as well as tract volume standardized to whole-brain volume (fNV). After exporting the DTI measures to SPSS, a multivariate analysis was used to compare FA, MD and fNV between HC and OB groups.

## Results

### Sociodemographic and clinical results

Sociodemographic information on the study sample is summarized in Table 1. No significant differences were found between groups with regards to age and years of education (*p* > 0.05). As expected, the OB group had significantly higher body fat percentage and BMI than the HC group (*p* < 0.001).

**Table 1:** Sample characteristics

	Healthy weight *n* = 25		Obese *n* = 24		
	Mean	SD	Mean	SD	*p*
Age (years)	31.92	12.95	38.08	10.43	0.099
Body mass index (BMI, kg/m^2^)	20.89	1.87	42.67	7.11	<0.001*
Body fat (%)	24.20	4.99	47.54	5.41	<0.001*
Years of education	15.68	1.60	14.92	2.00	0.179

*SD* standard deviation

*Bold: significant difference (*p* < 0.05). *p*-values include Finner’s correction for multiple comparisons

Participants in the OB group endorsed significantly greater difficulty in emotion regulation on the following DERS subscales (see Table S1): non-acceptance of emotional responses (*p* = 0.009), lack of emotion awareness (*p* = 0.001), and lack of emotional clarity (*p* = 0.049). Total DERS scores were also significantly higher in the OB group in comparison to the HC group (*p* = 0.048).

### Emotion regulation task results

#### In-scanner behavioral results

The analysis of in-scanner ratings for each condition (Observe, Maintain, and Regulate) revealed a main effect of condition (*F*(2, 94) = 60.94, *p* < 0.001). Post-hoc comparisons showed that Maintain differed from Observe, indicating successful negative emotion induction during this condition for both groups (Maintain > Observe: *F*(1, 47) = 95.84, *p* < 0.001). Likewise, comparisons between Regulate and Maintain contrasts showed significant differences between the two conditions (Maintain > Regulate *F*(1, 47) = 18.95, *p* < 0.001), indicating participants reduced distress levels when instructed to use cognitive reappraisal. No significant effects of group (*F*(1, 47) = 1.57, *p* > 0.05) or interactions (*p* > 0.05) were found.

#### Task activations and associations

**Fig. 1:**
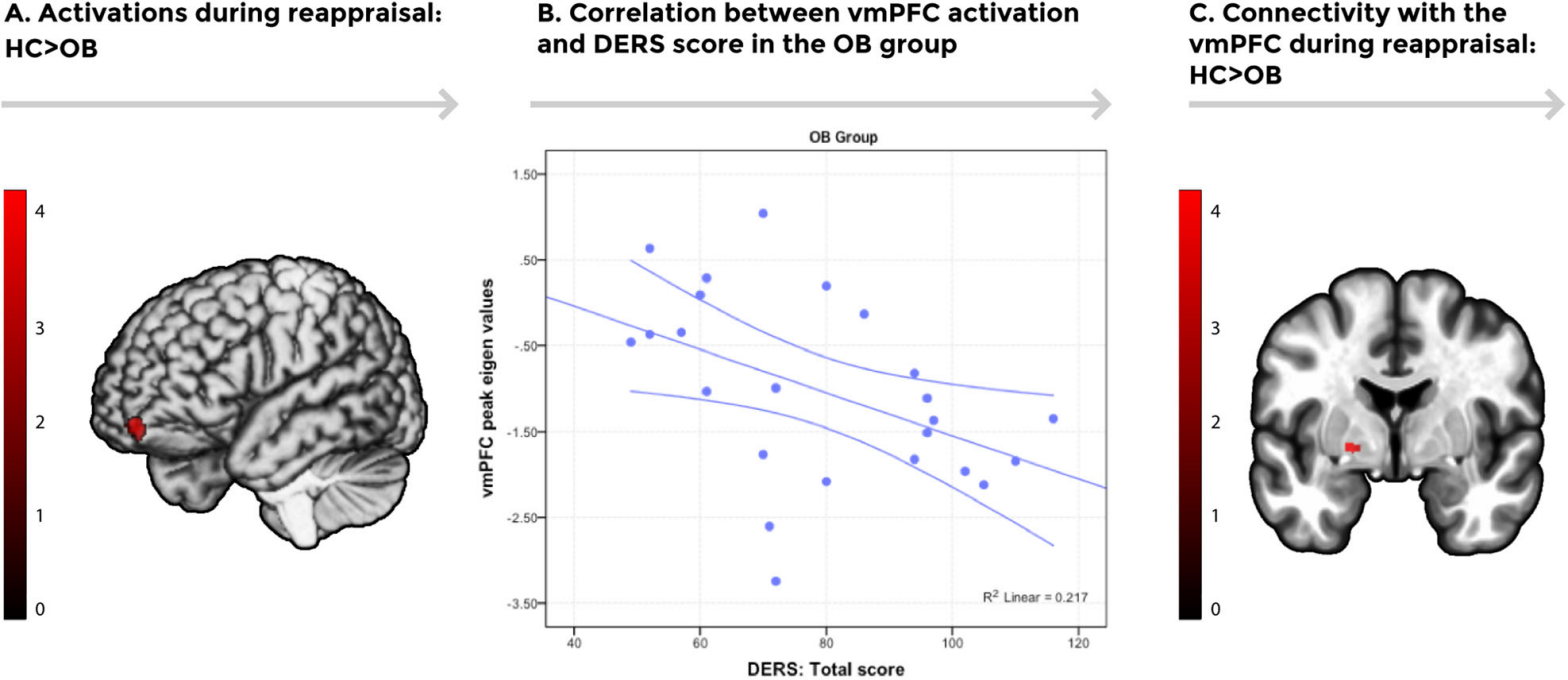
**a** Increased activation was found in the left ventromedial prefrontal cortex (vmPFC) in the healthy control group in comparison to the obese group (*p* < 0.05, AlphaSim cluster-extent corrected) during the emotion regulation task (Regulate vs. Maintain). Color bar represents *t*-values. **b** A scatterplot depicting the negative association between extracted activation eigenvalues from the vmPFC peak during the emotion regulation task (Regulate vs. Maintain) and Difficulties in Emotion Regulation Scale (DERS) total scores in the obesity group [*n* = 24, *r*(22) = −0.466, *p* = 0.022)]. Error bars show the 95% confidence interval. **c** Increased task-induced connectivity between the vmPFC and the left globus pallidus was found in the healthy control group in comparison to the obesity group using psychophysiological interactions (PPI) analysis (*p* < 0.05, AlphaSim cluster-extent corrected). Color bar represents *t*-values

**Fig. 2:**
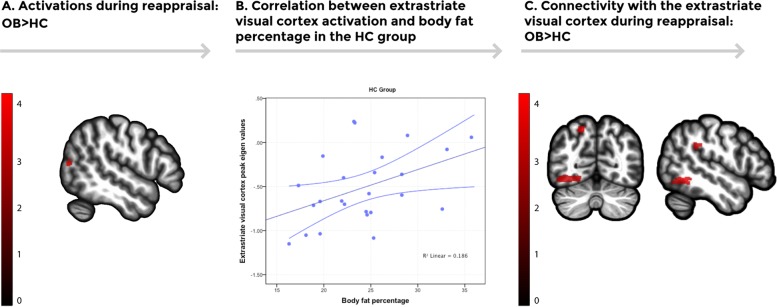
**a** Brain regions showing increased activation in the obese group during the emotion regulation task (Regulate vs. Maintain). Increased activation was found in the right extrastriatal visual cortex in the obese group in comparison to the HC group (*p* < 0.05, AlphaSim cluster-extent corrected). Color bar represents *t*-values. **b** A scatterplot depicting the positive correlation between extracted activation eigenvalues from the extrastriatal visual cortex peak during the emotion regulation task (Regulate vs. Maintain) and total body fat percentage in the control group [*n* = 25, (*r*(23) = 0.431, *p* = 0.031)]. Error bars show the 95% confidence interval (CI). **c** Increased psychophysiological interactions (PPI) connectivity using the extrastriatal visual cortex as a seed was found in the left inferior temporal lobe, the left precuneus, and the left supramarginal gyrus in the obese group in comparison to the healthy control group. Color bar represents *t*-values

No significant differences were found between groups during the Maintain > Observe contrast. Conversely, between-group comparisons showed that OB participants presented reduced activity in the left vmPFC compared to HC participants during the Regulate > Maintain contrast (Table 2, Fig. 1). In contrast, OB participants displayed increased activation in the right extrastriate visual cortex in comparison to HC in this same comparison (Table 2, Fig. 2).

**Table 2:** fMRI emotion regulation task results

Contrast	Peak region	MNI coordinates (*x*, *y*, *z*)	Ke^a^	*t*-statistic
HC > OB	Left ventromedial prefrontal cortex	−10, −64, −18	44	4.21
OB > HC	Right extrastriate visual cortex	56, −72, 10	35	4.50

Regions showing between-group differences during the Regulate > Maintain contrast of the emotion regulation task (AlphaSim voxel-level probability = *p* < 0.001, *p* < 0.05 FWE-cluster corrected)

*HC* healthy controls, *OB* obesity, *MNI* Montreal Neurological Institute

^a^Cluster extent in voxels

A negative association between the peak activation eigenvalues extracted from the vmPFC during Regulate vs. Maintain and DERS total scores was found in the study sample (*r*(47) = −0.397, *p* = 0.005) and in the OB group (*r*(22) = −0.466, *p* = 0.022). Likewise, a significant positive association was found between right extrastriate visual cortex peak eigenvalues during Regulate vs. Maintain and body fat percentage in the study sample (*r*(47) = 0.494, *p* < 0.005) and in the HC group (*r*(23) = 0.431, *p* = 0.031).

#### PPI connectivity

In comparison to HC, OB participants presented decreased connectivity between the left vmPFC and the left globus pallidus and increased connectivity between the vmPFC and the right crus cerebellum II (Table S2, Fig. 1). Increased connectivity was found between the right extrastriatal visual cortex and the left inferior temporal lobe, the left precuneus and the left supramarginal gyrus in the OB group compared to HC (Table S2, Fig. 2).

### Resting-state functional connectivity results

#### Resting-state vmPFC connectivity

**Fig. 3:**
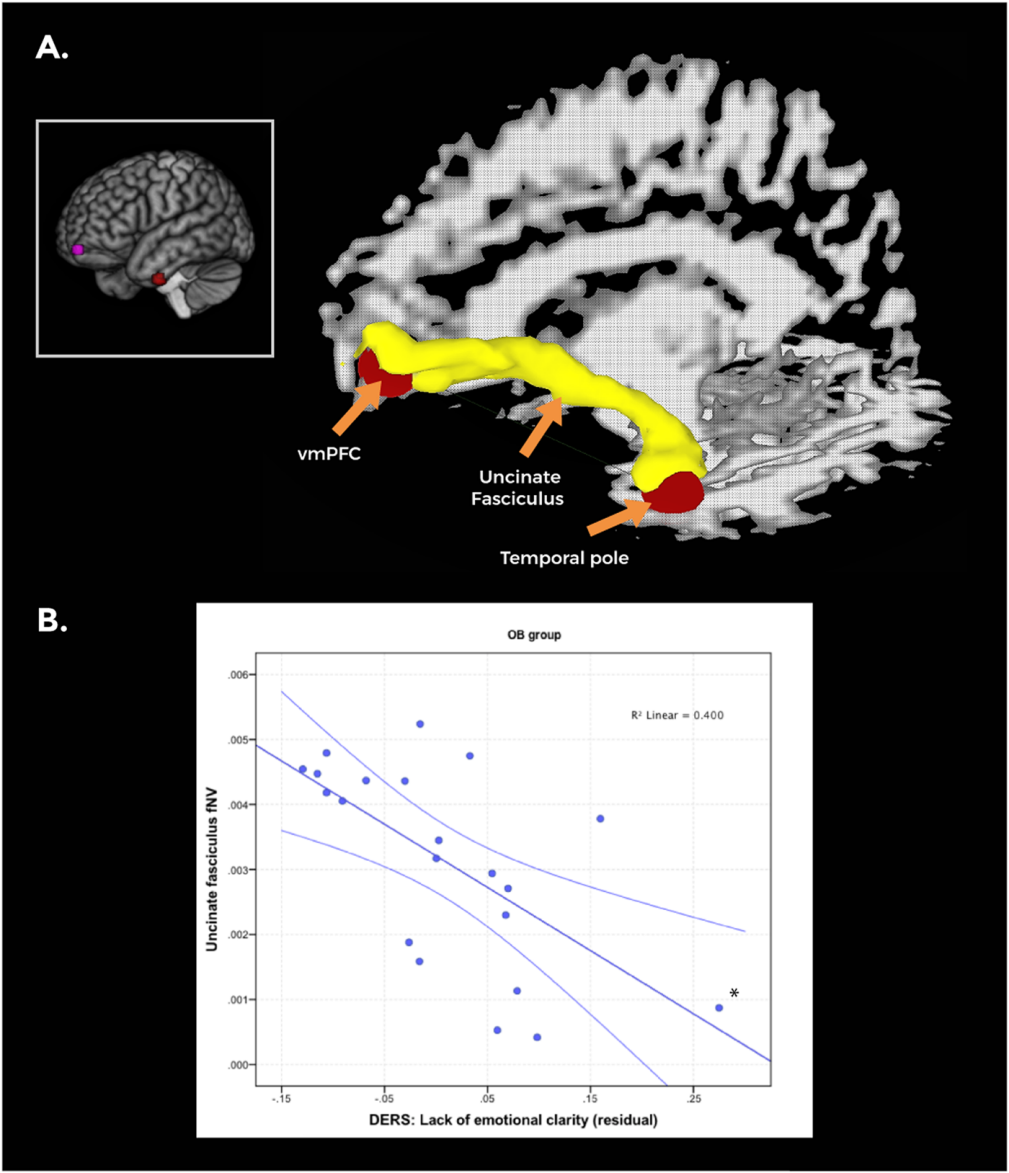
**a** Targets for probabilistic fiber tracking, derived from the resting-state analyses results (featured in the box), included the left ventromedial prefrontal cortex (vmPFC) and the left temporal pole (marked in red). An example of the uncinate fasciculus tracts connecting the vmPFC target with the temporal pole target is shown (marked in yellow). SUMA/AFNI Surface Mapper software was used for DTI image preparation. **b** A scatterplot depicting the negative correlation between the fractional volume of the left uncinate fasciculus and residual Difficulties in Emotion Regulation Scale (DERS) Lack of Clarity scores in the obese group controlling for age [*n* = 21, *r*(19) = −0.624, *p* = 0.002]. Error bars show the 95% confidence interval (CI). *This correlation remained significant after excluding the highlighted subject (*p* = 0.003 vs. *p* = 0.010)

Using the left vmPFC peak coordinate from the emotion regulation task for seed-to-voxel analysis, participants in the HC group, as compared to the OB group, presented increased intrinsic functional connectivity with the left temporal pole (peak-pFWE = 0.039, see Table S3, Fig. 3)

#### Resting-state extrastriate visual cortex connectivity

Participants in the OB group displayed increased functional connectivity during rest between the right extrastriate visual cortex and the left inferior temporal cortex compared to HC (cluster-pFWE = 0.016, Table S3, Fig. S1).

### DTI results

#### Distribution of tracts

White-matter tracts connecting the left vmPFC and the left temporal pole from the abovementioned resting-state analyses were identified in at least 85% of the total sample (*n* = 45). After visual inspection, and taking into account that our probabilistic tractography approach was implemented to isolate fibers between target ROIs in the vmPFC and temporal pole, individual white-matter tracts connecting these two regions were identified as belonging to the left uncinate fasciculus^38^. An example of identified uncinate fasciculus tracts from a representative subject can be found in Fig. 3. White-matter tracts connecting the right extrastriate visual cortex and the left inferior temporal lobe were found in an insufficient number of participants to be included in subsequent analyses.

#### Differences in DTI measures

A multivariate analysis comparing FA, MD and the fractional volume of tracks (fNV) between HC and OB participants was performed. Groups significantly differed in the measures examining left uncinate fasciculus microstructure (OB patients *n* = 21, HC *n* = 24, *F*(1, 42) = 3.56, *p* = 0.012). According to post-hoc tests, group differences were driven by lower fNV in the OB group compared to the HC group (*p* = 0.02) (Table 3).

**Table 3:** Comparison between groups in DTI white-matter microstructure measures

WM-ROI	DTI parameter	*F*-statistic	*p*-value	Direction
Left uncinate fasciculus	FA	0.11	0.744	−
	MD	3.27	0.077	−
	fNV	11.07	0.002	HC > OB

*WM-ROI* white-matter region of interest, *DTI* diffusion tensor image, *FA* fractional anisotropy, *MD* mean diffusivity, *fNV* fractional volume of tracts, *HC* healthy controls, *OB obese*

#### Associations between DTI and clinical measures

Within the whole sample [*r*(42) = −0.401, *p* = 0.006] and within the OB group [*r*(18) = −0.624, *p* = 0.002], fNV in the left uncinate fasciculus was negatively associated with DERS Lack of Emotional Clarity scores (Fig. 3).

## Discussion

The study at hand used multimodal MRI to identify the neurobiological foundation of altered emotion regulation in OB. During the completion of a cognitive reappraisal task, participants with OB displayed reduced activation of the vmPFC in comparison to HC participants. Additionally, activation levels in the vmPFC were negatively associated with self-reported difficulties in emotion regulation, suggesting that, despite being detected in an experimental context, the effects of vmPFC hypoactivation translate into real-life contexts. Human and animal studies have demonstrated that the vmPFC is a key node of cortical and subcortical networks subserving the generation and regulation of negative emotions via interactions with the amygdala, the dorsal anterior cingulate cortex and other regions^38,39^. By directly acting upon inhibitory interneurons within subcortical structures, the vmPFC mediates the inhibition of negative emotion and acts as a monitor for assessing the effectiveness of emotion regulation strategies^38–40^. Altered vmPFC activity during emotion regulation is commonly reported in populations with substance use disorders^41^, and with psychiatric disorders, such as mood and anxiety disorders^18,42^. Other work has implicated a failure to engage the vmPFC with loss-of-control eating in overweight and obese girls in response to distress, suggesting that perturbations in such neural circuits may lead to overeating in order to cope with negative affect^43^. More recent research using stress induction, food cues and ecological momentary assessment found that changes in activation in the vmPFC significantly moderated the relationship of affect to binge eating^44^, thereby lending support to models positing negative urgency as being a risk factor for bulimic symptoms^45^.

In this study, the specific subcortical structure with less functional connectivity from the vmPFC in the OB group when instructed to regulate negative emotions was the globus pallidus. As part of the cortico-striato-thalamo-cortical (CSTC) limbic loop, the globus pallidus plays a pivotal role in maintaining and regulating motivation by acting as a relay with the dorsomedial nucleus of the thalamus^46^. Disengagement of this loop in patients with OB when instructed to cognitively reappraise negative emotions may be suggestive of an inability to sustain an emotion regulation strategy for a sufficient length of time to effectively reduce negative emotions^47^. Moreover, this finding dovetails with the results of research showing that connectivity between the vmPFC and the striatum is crucial to cognitive reappraisal by shifting experienced emotional valence from negative to positive^48^. A disordered reward-attribution system, wherein the vmPFC fails to accurately attach value to stimuli, could constitute a general mechanism underpinning the difficulties reported by people with OB in resisting rewarding foods and wielding negative emotions^49^. Other studies including patients with OB and patients with binge eating disorder (BED) found dietary restraint scores to be negatively correlated with and vmPFC activity, supporting that BED individuals’ diminished ability to recruit impulse-control-related brain regions could be associated with impaired dietary restraint^50^.

Participants with OB presented decreased resting-state connectivity between the vmPFC and the left temporal pole, together with lower fractional volume in the white-matter tracts connecting these regions (the left uncinate fasciculus). Moreover, fNV values from the uncinate fasciculus were negatively associated with DERS Lack of Emotional Clarity scores, indicating that lower uncinate tract volume could underlie the dispositional difficulties in this specific dimension of emotion regulation. One of the overarching functions of the uncinate fasciculus is to allow mnemonic representations originating in the temporal lobe to modify behavior through interactions with prefrontal regions supplying valence-based biasing of decisions^38^. Similarly, uncinate fasciculus tracts between the anterior temporal lobe and the prefrontal cortex also pass through the amygdala and are understood to contribute to the emotional tone (i.e. positive or negative feelings, and personal significance) of representations stored in the anterior temporal lobe^51^. Disruption of the uncinate is believed to cause problems in guiding decisions and to underlie numerous psychiatric disorders^52,53^. It is likely that uncinate perturbation could potentially be problematic in domains that extend beyond memory to include social–emotional functioning and higher-level motivation^38^. Overall, our findings lend support to the notion that, beyond the impairments demonstrated by individuals with OB when confronting negative scenarios, specific trait-like features leading to impaired emotion regulation capacities, such as difficulties correctly identifying emotions^6,54,55^, may be at least partly underpinned by alterations in circuits not necessarily engaged during attempts of emotion regulation in experimental settings.

The OB group presented differential activation patterns in the extrastriate visual cortex in comparison to controls during both cognitive reappraisal and at rest. The extrastriate visual cortex is highly implicated in the conscious perception of emotional signals and its response is known to be amplified by stress in a manner consistent with vigilance for threat^56,57^. For example, the extrastriate body area (EBA), which is part of our pattern of findings, is known to be involved in the processing of emotionally salient stimuli^58^. Hyperactivation of extrastriate visual areas in OB patients, and increased connectivity of this region with other high-order visual processing areas, could be indicative of diminished control in the visual regions while processing emotionally salient stimuli^59^. In this sense, bidirectional feedback connections between the amygdala and extrastriatal visual areas are involved in the perceptual enhancement of emotionally relevant stimuli^60^ and failure to properly engage this visual processing stream is likely to underlie in some measure the attentional biases found in OB^60–62^. Importantly, activation in the extrastriate visual cortex during emotion regulation was positively associated with body fat percentage in the control group, which indicates that increased activation of this region is not exclusively observed in obese individuals but also in those lean controls with a higher body fat percentage. Further research should aim at ascertaining the putative mechanisms linking adiposity to overreaction to affective stimuli. Body fat has indeed been shown to modulate activity in other brain networks, such as those subserving homeostatic and reward signal processing^63^.

Interpretations from the presented findings should be made bearing in mind the limitations of this study. First, its cross-sectional design does not allow for inferences about causality. In this sense, it would be of great interest to examine how emotion regulation deficits may pose a risk factor for developing OB^64^. Second, we recruited a moderately sized sample of individuals with OB, though we did aim for homogeneity in that the sample was made up of only women, since sex has been shown to be a significant confounder associated with emotion dysregulation^65^. As a final limitation, no quantitative data on the nutritional aspects of the participants’ eating behaviors were collected. Future studies would stand to benefit from assessing the extent to which neural activation patterns during emotion regulation are linked to the consumption of highly palatable “comfort” foods.^23^

## Conclusion

This study advances our understanding of the neural foundation of emotion regulation impairments in OB. Using a multimodal MRI approach, we demonstrated that hypoactivation in the vmPFC during emotion regulation is associated with self-reported emotion regulation difficulties in patients with OB. Furthermore, we identified how structural and functional connectivity alterations originating in the vmPFC and spanning the uncinate fasciculus are linked to excess weight and emotion regulation deficits, thereby providing researchers and clinicians alike with a potential target circuit for neuromodulatory interventions^66^.

## Supplementary information

Supplementary Material
