# Supplementary material for: A multimodal MRI study of the neural mechanisms of emotion regulation impairment in women with obesity
Source: Transl Psychiatry. 2019 Aug 20;9:194. doi: 10.1038/s41398-019-0533-3 (PMC6702163; doi:10.1038/s41398-019-0533-3)
Supplement: Supplementary file 1 — Supplementary Material [file 41398_2019_533_MOESM1_ESM.docx]

Supplementary Information

A Multimodal MRI Study of the Neural Mechanisms of Emotion Regulation Impairment in Women with Obesity

Trevor Steward, Maria Picó-Pérez, Gemma Mestre-Bach, Ignacio Martínez-Zalacaín, Maria Suñol, Susana Jiménez-Murcia, Jose A Fernández-Formoso, Nuria Vilarrasa; Amador García-Ruiz-de-Gordejuela; Misericordia Veciana de las Heras; Nuria Custal; Nuria Virgili; Rafael Lopez-Urdiales; José M Menchón, Roser Granero, Carles Soriano-Mas, Fernando Fernandez-Aranda

Email: csoriano@idibell.cat & ffernandez@bellvitgehospital.cat

**This file includes:**

Supplementary text

Fig. S1

Tables S1 to S3

References for SI reference citations

Supplementary Information Text

**Inclusion and exclusion criteria**

The study inclusion criteria were the following: being female and being between the age of 18 and 55. Healthy control (HC) participants were required to have a body mass index (BMI) between 18.5 and 25.0. The study exclusion criteria were: being male, the presence of an organic mental disorder or an intellectual disability, a current eating disorder or another psychiatric disorder (psychotic disorders, bipolar disorder, substance dependence, or anxiety and depressive disorders). Prior to assessment, HC participants were asked to report maximum lifetime BMI and those who endorsed having had obesity (BMI>30) were excluded from the study sample. HC participants received compensation (€80) for participating in the study.

**MRI image acquisition**

Participants were scanned using a 3T Phillips Ingenia system (Philips Medical Systems) equipped with a thirty-two-channel phased-array head coil. Two functional MRI (fMRI) sequences (i.e., one during resting-state and another during the completion of the emotion regulation task, see below) were acquired with identical parameters: a single-shot gradient-echo echo-planar imaging (EPI) was used. This sequence feature: repetition time, 2000 msec; echo time, 25 msec; and pulse angle, 90°; in a 24-cm field of view; and an 80 × 80-pixel matrix; providing isotropic voxel sizes of 3 × 3 x 3 mm, with no gap. 40 interleaved sections, parallel to the anterior-posterior commissure line, were acquired for each whole-brain volume. For the resting-state time-series, we acquired 240 volumes (8:00 minutes), whereas for the emotion regulation task we acquired 234 volumes (7:48 minutes).

Diffusion-weighted scans were also obtained using spin-echo single-shot echo-planar sequences with 64 directions and a b-value of 1000 s/mm2, together with a single non–diffusion weighted volume. Fifty slices were acquired parallel to the anterior-posterior commissure (repetition time, 4970 msec; echo time, 65 msec; and pulse angle, 90°) in a 24-cm field of view, with a 96 x 96 acquisition matrix and isotropic voxel sizes of 2.5 x 2.5 x 2.5 mm.

Finally, a high-resolution T1-weighted anatomical scan was also acquired to facilitate registration of EPI and diffusion-tensor imaging (DTI) data into standard space. Specifically, we used a three-dimensional fast-spoiled gradient, inversion-recovery sequence with 233 contiguous slices (repetition time, 10.43 msec; echo time, 4.8 msec; flip angle, 8°) in a 24-cm field of view, with a 320 × 320 pixel matrix and isotropic voxel sizes of 0.75 × 0.75 x 0.75 mm.

**fMRI image preprocessing**

All functional images (resting-state and task-based) were initially preprocessed using the Wavelet Despike procedure within the BrainWavelet Toolbox (1). This process removes a range of high and low frequency artifacts from an fMRI time series by denoising synchronized signal transients induced by abrupt physical movements. In addition to these movement parameters, white matter, cerebrospinal fluid (CSF), and global blood oxygen level-dependent (BOLD) time-series were also introduced as confounders in a further denoising stage performed with the CONN toolbox (version 17.f, McGovern Institute for Brain Research, Massachusetts Institute of Technology, Cambridge, USA, http://www.nitrc.org/projects/conn).

Image preprocessing was performed using statistical parametric mapping software (SPM 12, Wellcome Department of Imaging Neuroscience, London, England; www.fil.ion.ucl.ac.uk/spm) and CONN toolbox running on MATLAB R2017a. Each subject’s functional images were realigned to the mean position of all scans and high-resolution structural T1 images were oriented to the anterior and posterior commissure (AC-PC) line. Functional scans were then co-registered to their respective T1 images, which were used for normalization to the standard Montreal Neurological Institute (MNI) space. Normalization parameters were then applied to functional time-series, which were finally smoothed with an 8-mm full width at half maximum (FWHM) kernel.

**Emotion regulation task**

Prior to scanning, participants were given instruction on how to utilize reappraisal strategies. Three types of reinterpretations were recommended to participants using an example situation: (1) the scene is not real (e.g. the people on the screen are actors); (2) the situation will likely get better with time; and (3) the situation is not as grave as it first appears (e.g. seeing the situation in a more positive light). Participant were specifically instructed that they were not to use non-cognitive strategies (i.e. as looking away) during stimulus presentation.

Each block began with the instructive prompt (Observe, Maintain or Regulate) presented in the middle of the screen for four seconds. After the prompt, participants viewed two different pictures of equal valence for ten seconds each. After the presentation of the second picture of each block, the intensity of the participants’ distress was self-rated on a 1–5 numeric scale (1 being ‘neutral’ and 5 being ‘extremely negative’) in order to confirm whether participants were successfully carrying out the task instructions. Interactions between in-scanner ratings for each condition (Observe, Maintain and Regulate) and subject groups were evaluated using a 2x3 repeated-measures ANOVA analysis.

24 images from the International Affective Picture System (2) were used for the task: eight neutral pictures (e.g. household objects), which were presented in the Observe condition, and 16 highly unpleasant pictures (e.g. mutilations) in the Maintain and Regulate conditions. In total, the task consisted of twelve, 20-second blocks including the presentation of two images for ten seconds, with a total of four blocks for each condition. Instructions (Observe, Maintain or Regulate) were pseudo-randomized throughout the task to avoid the induction of sustained mood states. In-scanner ratings were recorded through an fMRI-compatible response pad (Lumina–Cedrus Corporation). Each block was followed by a 10-second presentation of a cross fixation to separate each block and to minimize carry-over effects. Task instructions and visual stimuli were presented using Presentation® software (Version 18.3, build 03.11.16, www. neurobs.com) through an MRI-compatible BOLD screen (BOLDscreen 32, Cambridge Research Systems) located at the rear of the scanner gantry. An angled mirror system was used to allow participants to view the images.

Images were selected according to International Affective Picture System normative values for valence and arousal; mean valence values were 5.79 (0.71), 2.53 (0.69), 2.66 (0.68) and mean arousal values were 4.28 (0.73), 6.44 (0.46) and 6.40 (0.60) for images included in the Observe, Maintain, and Regulate conditions, respectively (2). Pairwise comparisons showed that Maintain and Regulate did not differ in valence or arousal (P>0.7), whereas both differed from Observe values (P<0.001).

Fig. S1.

a)


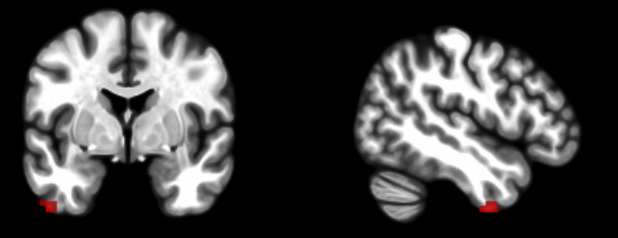

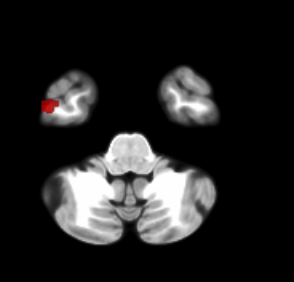


b)


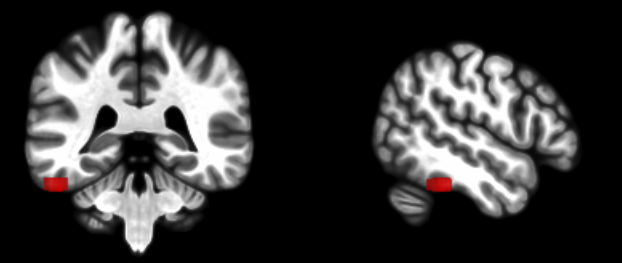

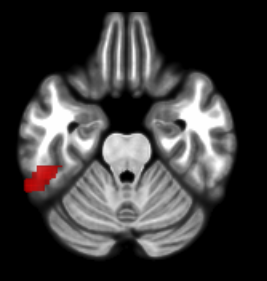


Regions showing between-group differences in functional connectivity during resting state. a) Using the left vmPFC peak coordinate (MNI: -10, -64, -18) from the emotion regulation task for seed-to-voxel analysis, participants in the HC group presented increased functional connectivity with the left temporal pole in comparison to the OB group (peak-pFWE=0.039; b) Using the right extrastriate visual cortex peak coordinate (MNI: 56, -72, 10) from the emotion regulation task, participants in the OB group presented increased functional connectivity with the left inferior temporal cortex compared to HC (cluster-pFWE=0.016).

**Supporting Tables**

**Table S1.** Comparison of Difficulties in Emotion Regulation (DERS) scores in the study sample.

|  |  | Healthy weight  *n=25* | | Obese  *n=24* | |  |  |  | |
| --- | --- | --- | --- | --- | --- | --- | --- | --- | --- |
|  | α | *Mean* | *SD* | *Mean* | *SD* | *p* | *\|d\|* | *Power* |  |
| DERS: Non-acceptance of emotional responses | .839 | 10.56 | 3.59 | 14.92 | 6.27 | **.009*** | **0.85^†^** | 0.78 |  |
| DERS: Difficulty engaging in goal-directed behavior | .715 | 12.00 | 3.66 | 11.54 | 3.65 | .688 | 0.13 | 0.25 |  |
| DERS: Impulse control difficulties | .664 | 9.32 | 2.64 | 10.38 | 3.23 | .245 | 0.36 | 0.16 |  |
| DERS: Lack of emotional awareness | .670 | 12.72 | 3.52 | 16.75 | 3.89 | **.001*** | **1.09^†^** | 0.74 |  |
| DERS: Limited access to emotion regulation strategies | .886 | 15.56 | 6.08 | 15.25 | 6.00 | .858 | 0.05 | 0.06 |  |
| DERS: Lack of emotional clarity | .904 | 8.16 | 2.88 | 10.46 | 4.46 | **.049*** | **0.61^†^** | 0.50 |  |
| DERS: Total score | .953 | 68.32 | 14.28 | 79.29 | 19.90 | **.048*** | **0.63^†^** | 0.72 |  |

*Note.* α*: Cronbach’s alpha in the study sample. SD: standard deviation.*

**Bold: significant difference (p<.05). †Effect size in the moderate (|d|>0.50) to high range (|d|>0.80).*

*p-values include Finner’s correction for multiple comparisons.*

**Table S2.** Emotion regulation functional connectivity psychophysiological interaction (PPI) results.

| **ROI** | **Contrast** | **Peak region** | **MNI coordinates**  **(x, y, z)** | **Ke^a^** | **t-statistic** |
| --- | --- | --- | --- | --- | --- |
| Left vmPFC | HC>OB | Left globus pallidus | -22, -2, -2 | 14 | 4.22 |
|  | OB>HC | Right crus cerebellum II | 12, -84, -30 | 38 | 3.64 |
| Right extrastriatal visual cortex | OB>HC | Left inferior temporal lobe | -48, -56, -8 | 841 | 5.07 |
|  |  | Left precuneus | -18, -68, 54 | 77 | 4.40 |
|  |  | Left supramarginal gyrus | -58, -26, 44 | 66 | 3.92 |

Regions showing between-group differences in functional connectivity during the Regulate>Maintain contrast of the emotion regulation task (AlphaSim voxel-level probability = p < 0.001, p < 0.05 FWE-cluster corrected). *MNI:* Montreal Neurological Institute, *ROI:* region of interest, *HC:* healthy controls, *OB: obesity*. ^a^Cluster extent in voxels.

**Table S3.** Resting-state seed-to-voxel functional connectivity results.

| **ROI** | **Contrast** | **Peak region** | **MNI coordinates**  **(x, y, z)** | **Ke^a^** | **t-statistic** |
| --- | --- | --- | --- | --- | --- |
| Left vmPFC | HC>OB | Left temporal pole | -50, -02, -46 | 67 | 5.56* |
| Right extrastriatal visual cortex | OB>HC | Left inferior temporal lobe | -60, -46, -26 | 255 | 5.45** |

Regions showing between-group differences in functional connectivity during resting state. *peak-voxel p-FWE corrected = 0.039; **cluster-size p-FWE corrected = 0.016; *HC*: healthy controls; *OB*: obesity; *MNI*: Montreal Neurological Institute *ROI:* region of interest. ^a^Cluster extent in voxels.

**References**

1. Patel AX, et al. (2014) A wavelet method for modeling and despiking motion artifacts from resting-state fMRI time series. *Neuroimage* 95:287–304.

2. Lang PJ, Bradley MM, Cuthbert BN (2008) *International affective picture system (IAPS): Instruction manual and affective ratings.* doi:10.1016/j.epsr.2006.03.016.
